# Chemical Mutagenesis and Fluorescence-Based High-Throughput Screening for Enhanced Accumulation of Carotenoids in a Model Marine Diatom Phaeodactylum tricornutum

**DOI:** 10.3390/md16080272

**Published:** 2018-08-04

**Authors:** Zhiqian Yi, Yixi Su, Maonian Xu, Andreas Bergmann, Saevar Ingthorsson, Ottar Rolfsson, Kourosh Salehi-Ashtiani, Sigurdur Brynjolfsson, Weiqi Fu

**Affiliations:** 1 Center for Systems Biology and Faculty of Industrial Engineering, Mechanical Engineering and Computer Science, School of Engineering and Natural Sciences, University of Iceland, 101 Reykjavík, Iceland; zhy1@hi.is (Z.Y.); yis2@hi.is (Y.S.); andreasbergmannhome@gmail.com (A.B.); ottarr@hi.is (O.R.); sb@hi.is (S.B.); 2 Biomedical Center and Department of Anatomy, Faulty of Medicine, University of Iceland, 101 Reykjavík, Iceland; saevari@hi.is; 3 Faculty of Pharmaceutical Sciences, University of Iceland, Hagi, Hofsvallagata 53, 107 Reykjavik, Iceland; maonian@hi.is; 4 Division of Science and Math, and Center for Genomics and Systems Biology (CGSB), New York University Abu Dhabi, Abu Dhabi 129188, UAE; ksa3@nyu.edu

**Keywords:** mutagenesis, screening, diatom, EMS, carotenoids, fucoxanthin

## Abstract

Diatoms are a major group of unicellular algae that are rich in lipids and carotenoids. However, sustained research efforts are needed to improve the strain performance for high product yields towards commercialization. In this study, we generated a number of mutants of the model diatom *Phaeodactylum tricornutum*, a cosmopolitan species that has also been found in Nordic region, using the chemical mutagens ethyl methanesulfonate (EMS) and *N*-methyl-*N*′-nitro-*N*-nitrosoguanidine (NTG). We found that both chlorophyll *a* and neutral lipids had a significant correlation with carotenoid content and these correlations were better during exponential growth than in the stationary growth phase. Then, we studied *P. tricornutum* common metabolic pathways and analyzed correlated enzymatic reactions between fucoxanthin synthesis and pigmentation or lipid metabolism through a genome-scale metabolic model. The integration of the computational results with liquid chromatography-mass spectrometry data revealed key compounds underlying the correlative metabolic pathways. Approximately 1000 strains were screened using fluorescence-based high-throughput method and five mutants selected had 33% or higher total carotenoids than the wild type, in which four strains remained stable in the long term and the top mutant exhibited an increase of 69.3% in fucoxanthin content compared to the wild type. The platform described in this study may be applied to the screening of other high performing diatom strains for industrial applications.

## 1. Introduction

Diatoms are a major group of unicellular algae, and they play a vital role in global ecosystems [1]. They are responsible for nearly half of the primary production and represent the base for marine food webs [1], and various products from marine diatoms such as pigments, polyunsaturated fatty acids (PUFAs) and neutral lipids (biodiesels) have attracted extensive attention, as their commercialization has been exploited in recent years [1,2,3]. *Phaeodactylum tricornutum* Bohlin, a model diatom species, has been widely studied due to its ease of cultivation and fully sequenced genome [1]. Although *P. tricornutum* is not usually considered as a widely distributed species, it has been found in many European countries including Finland [4].

One of the most valuable metabolites in *P. tricornutum* is fucoxanthin, a xanthophyll pigment that is one of the most abundant carotenoids. It is localized primarily in the chloroplast and binds to chlorophyll *a*/*c* to form fucoxanthin-chlorophyll-binding proteins (FCPs), which play a major role in light-harvesting systems [5,6]. The remarkable biological traits of fucoxanthin are based on its molecular structure, which contains one allenic bond and a few oxygenic functional groups such as hydroxyl, carbonyl and epoxy moieties [7]. This pigment has numerous health-promoting effects such as antioxidant, anti-inflammation, anticancer, anti-obesity, and antidiabetic activities [7].

Biotechnological methods such as genetic modifications and mutagenesis have been developed to improve strain characteristics and the production of valuable alga-derived products [3,8,9,10]. Briefly, genetic engineering is a rational approach where selected genes are manipulated. The approach has been applied to diatoms to increase the yield of value-added products such as carotenoids and fatty acids [9]. On the other hand, mutagenesis leads to random changes in the genome, resulting in unpredictable outcomes: while, in most cases, randomized mutagenesis is likely to create mutants with lower yields than the wild type (WT), rare random mutations with a positive effect can be isolated if an effective screening strategy is used [11]. Thus, diverse mutagenesis experiments have increased the yields of target products in different studies [12,13]. Since the 1990s, most studies on engineering microalgae to overproduce carotenoids were conducted by screening for mutants that could resist carotenogenic pathway inhibitors such as nicotine, norflurazon, glufosinate, diphenylamine (DPA) and compactin [14,15,16,17]. The herbicide-resistant mutants are expected to possess mutated enzymes with altered expression and enzymatic properties that enable the synthesis of desired pigments in the presence of inhibitors and certain herbicides as nicotine and DPA were known to inhibit lycopene cyclase [12]. An advantage of random mutagenesis is its simplicity, as it requires little knowledge of the biosynthetic pathways of the desired products and very few technical operations [12]. More importantly, the resulting mutants are not subject to regulations for genetically modified organisms (GMOs) [18] in the food industry in Europe and many other regions. In situations where limited molecular tools are available for microalgal genetic engineering, random mutagenesis can be an important approach for developing improved algal strains [18]. 

Depending on the mutagen properties, mutagenesis can be divided into physical and chemical mutagenesis. Physical mutagens contain electromagnetic radiation such as X-rays, UV light and particle radiation as β and α particles [19]. Among chemical mutagens, alkylating agents such as ethyl methanesulfonate (EMS) and *N*-methyl-*N*′-nitro-*N*-nitrosoguanidine (NTG) are the most widely used for creating positive algal mutants with high lipid or carotenoid content [12]. The genomes have been randomly modified by mutagenesis, which sometimes leads to various phenotypic effects. The diploid nature of vegetative *P. tricornutum* cells could lead to carotenoid content fluctuations in mutants because the allele for a particular gene in one chromosome chain may be positively mutated while the other one might not [20,21].

Conventional methods for screening mutated strains with high carotenoid content require manual inspection of every colony, which is time-consuming and inefficient [22]. After many mutated strains have been created, efficient screening of the desired phenotypes is the critical step and a major bottleneck in mutagenesis applications. In the green alga *Dunaliella salina salina* (Dunal) Teodoresco, a flow cytometry-based approach has been used to examine correlations between lipidic composition and carotenoids for establishing a high-throughput screening method [22], while little information is presently available for diatoms. 

Among abundant computational approaches, genome-scale network reconstructions are a crucial part in connecting genome information to phenotypes. One promising strategy for deciphering undiscovered potential correlations between certain creatures is metabolic network reconstruction, which could be utilized to analyze system level reactions [23]. A comprehensive genome-scale metabolic model of *P. tricornutum* was recently published [23]. It is based on genomic, genetic, and biochemical knowledge and includes information on connections between genes and reactions as well as reaction stoichiometry. Genome-scale models (GEMs) enable exploration of the complex diatomic metabolism via quantitative predictions. 

**Figure 1:**
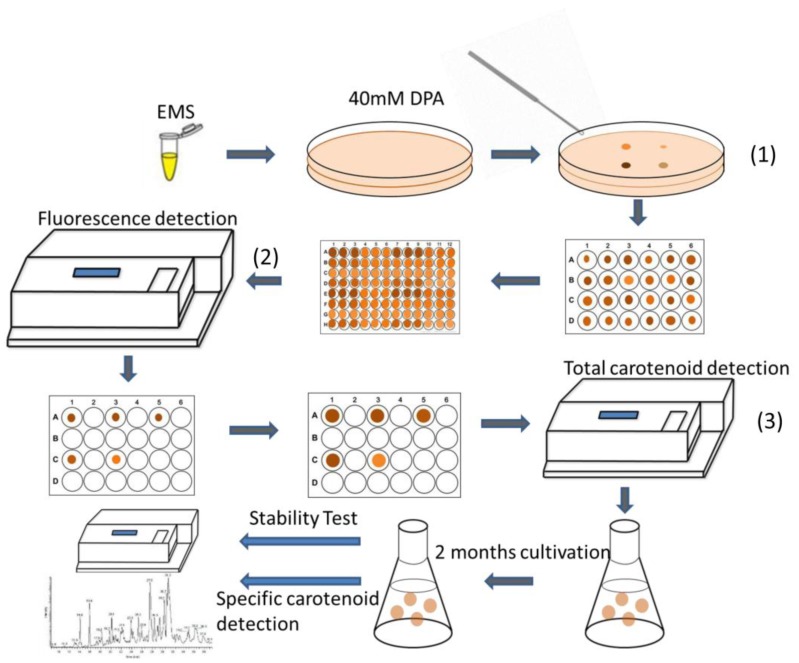
Schematic process for high-throughput screening of targeted mutants. The detailed description was in the Experimental Section. There were three main screening steps for this method: (**1**) select colonies with large size and deep color for microplate cultivation; (**2**) pick out strains with relatively high chlorophyll *a* and Nile red fluorescence intensity; (**3**) select strains with high total carotenoid content following with pigment extraction.

In this study, we are aiming at creating a high-throughput method to increase screening efficiency of selecting fucoxanthin-hyperproducing strains from mutagenesis. We first applied DPA as an inhibitor of the carotenogenic pathway and tested its effects on *P. tricornutum* growth. Then, we compared the mutagenesis effects of EMS as well as NTG on *P. tricornutum.* Under similar lethality rate, EMS showed a higher efficiency for creating positive mutants with higher carotenoid content. In addition, as we found that both chlorophyll *a* and total neutral lipid fluorescence intensity had significant correlations with carotenoid metabolism, we established a high-throughput screening method (Figure 1); five mutants were selected from 1000 isolated strains based on this method. Mutants were cultivated over two months to validate strain stability, and liquid chromatography-mass spectrometry (LC-MS) was applied to detect specific lipophilic compounds. Finally, four of five selected diatom mutants exhibited higher fucoxanthin production than the WT strain.

## 2. Results 

### 2.1. Effect of Different Doses of DPA on P. tricornutum Growth

Herbicides have been widely applied in mutagenesis experiments to create mutants with higher yields of targeted products [12]. DPA can inhibit carotenoid synthesis [12], and the purpose of applying DPA in this study was to enhance the selective pressure for isolating positive mutants, as DPA-resistant mutants will likely have higher fucoxanthin contents. It was found that 10 μM DPA treatment of the WT reduced the specific growth rate from 0.645 day^−1^ to 0.431 day^−1^ (Figure S2). When DPA was applied in a range from 30 μM to 60 μM, the diatom specific growth rate decreased significantly. Particularly, the WT still grew when the DPA concentration was below 40 μM, but cell numbers declined once the DPA concentration exceeded 40 μM. Consequently, 40 μM DPA was chosen for the subsequent screening experiments.

### 2.2. Effects of EMS and NTG on Creating Positive Mutants

**Figure 2:**
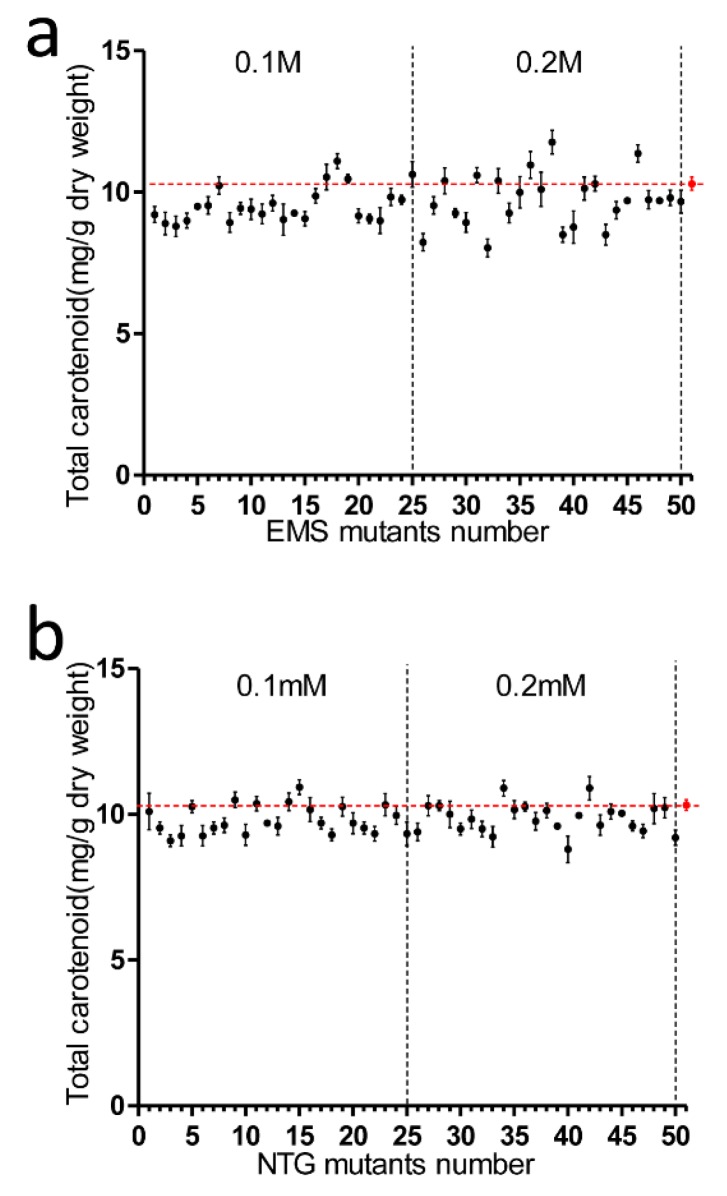
Analysis of total carotenoid content in EMS and NTG mutants. (**a**) EMS mutagenesis, from the *y*-axis to left dotted line are No. 1–No. 25 mutants treated with 0.1 M EMS, No. 26–No. 50 mutants were treated with 0.2 M EMS; (**b**) NTG mutagenesis, mutants No. 1 to No. 25 were treated with 0.1 mM NTG while No. 26 to No. 50 mutants were treated with 0.2 mM NTG. The strain designated as No. 51 is the untreated wild type; the transverse dotted line represented wild type total carotenoid concentration. Each data point corresponds to the average value from triplicate experiments.

We examined the ability of both EMS and NTG to create *P. tricornutum* mutants. As fucoxanthin was the dominant carotenoid in *P. tricornutum*, the total carotenoid amount could be utilized as a good indicator for fucoxanthin content. The total carotenoid content of mutants in the 0.1 M EMS group varied from 8.8 to 11.1 mg/g DW (Figure 2a). Two mutants in the 0.1 M EMS group had higher carotenoid content than WT (10.3 mg/g), but neither mutant’s content exceeded that of the WT by more than 10%. For the 0.2 M EMS group, total carotenoid content varied from 8.0 to 11.8 mg/g DW. Four mutants had higher carotenoid content than WT, and two mutants among these had more than 10% total carotenoids greater than WT. For the 0.1 mM NTG group, total carotenoid content varied from 9.1 to 10.9 mg/g DW; three mutants had higher total carotenoids than WT, but all the differences between mutant and WT carotenoid content were less than 10% (Figure 2b). In the 0.2 mM NTG group, the carotenoid content varied from 8.8 to 11.1 mg/g DW. Three mutants had higher carotenoid content than WT, but the differences between mutant and WT content were all under 10%. The cell lethality of 0.1 M and 0.2 M EMS in diatoms was 42.3% and 71.5%, respectively, while 0.1 mM and 0.2 mM NTG caused 36.9% and 65.8% death rates. It implied that EMS had better efficiency than NTG at similar lethality rates of creating carotenoid hyper-production mutants in *P. tricornutum* at both concentrations. EMS was chosen for the following mutagenesis procedures.

### 2.3. Correlations of Both Chlorophyll a and Lipids with Carotenoid Metabolism

**Figure 3:**
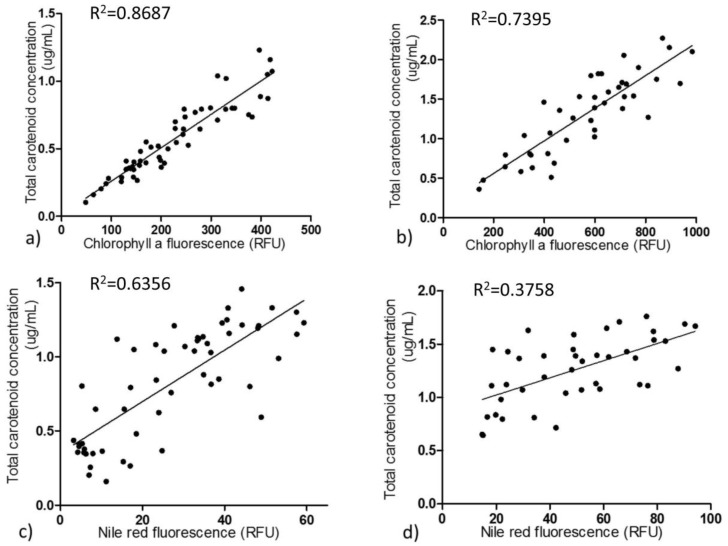
Correlation of chlorophyll *a* fluorescence and Nile red fluorescence with total carotenoid content. Correlation between chlorophyll *a* fluorescence intensity and total carotenoid content in *P. tricornutum* in exponential (**a**) and stationary (**b**) growth phases, respectively. Correlation between Nile red fluorescence intensity and total carotenoid content in exponential (**c**) and stationary growth (**d**) phases, respectively. Each dot represents the averaged value of each strain from biological triplicates. Chlorophyll *a* and Nile red fluorescence were measured in 96 well plates by a fluorescence spectrophotometer. All four of these correlations are significant (*p* < 0.01).

As carotenoid fluorescence was relatively low and chlorophyll *a* fluorescence was higher and is easily detected [22], we tested the correlations between chlorophyll *a* fluorescence intensity and total carotenoid content in order to develop an effective and quick method to screen fucoxanthin-rich mutants (Figure 3). During the exponential growth phase, chlorophyll *a* exhibited a good linear correlation with total carotenoid content with 0.8687 coefficient value. The relationship between chlorophyll *a* content and carotenoids and their corresponding coefficient of determination are provided (Table S1). During the stationary growth phase, the correlation was not as good as in the exponential state. Nile red, as a lipophilic dye that integrates into intracellular lipids, can irradiate strong fluorescence under excitation at 530 nm [24]. Nile red fluorescence intensity correlates linearly with cellular neutral lipid content [24]; therefore, Nile red fluorescence was utilized to explore the relations between neutral lipid composition and total carotenoids. In the exponential growth phase, Nile red fluorescence intensity and total carotenoids also had a moderately linear correlation with coefficient value 0.6356. Nevertheless, the correlation between Nile red fluorescence intensity and total carotenoids was much lower in the stationary phase (Table S1).

### 2.4. Detection and Analysis of Major Pigments and Lipids in the Diatom Dtrains

**Figure 4:**
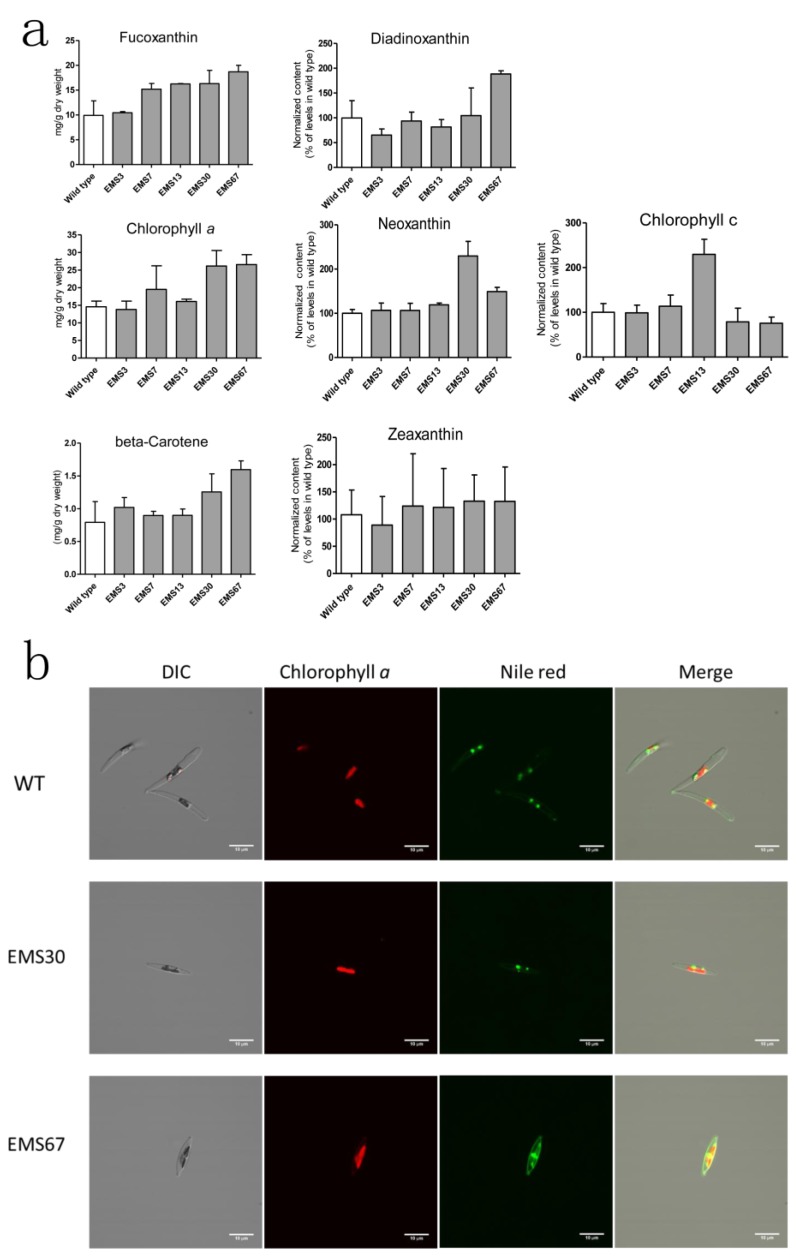
Analysis of pigments and lipids in wild type and selected positive mutants. (**a**) both wild type and mutants were analyzed during the exponential growth phase. Pigments were extracted and determined using ultrahigh performance liquid chromatography-mass spectrometry (UPLC-MS), reported values are the averages of three biological replicates; (**b**) chlorophyll *a* and Nile red fluorescence observed through a confocal microscope. Intergroup comparison was conducted by one-way ANOVA, scale bar = 10 μM.

Five positive mutants screened by the high-throughput process were selected for LC-MS analysis, and seven pigments were quantitated. As shown in Figure 4a, EMS7, EMS13, EMS30 and EMS67 strains exhibited significantly higher fucoxanthin content than WT, while EMS3 had similar content as WT (based on one-way ANOVA analysis for fucoxanthin content in each strain). Among these five mutants, EMS67 had the highest fucoxanthin accumulation, 69.3% higher than that of WT, while EMS7, EMS13 and EMS30 fucoxanthin contents were 53.2%, 63.8% and 64.2% greater than that of WT, respectively. For chlorophyll *a*, four of five mutants had greater accumulation than WT; EMS7, EMS13, EMS30 and EMS67 had 33.7%, 10.2%, 79.1% and 81.9% more than WT, respectively, while EMS3 displayed similar content to WT. For beta-carotene, all five mutants showed higher accumulation than WT; EMS67 had 101.5% more beta-carotene than WT. EMS30 and EMS67 had 129.5% and 49.1% more neoxanthin than WT, respectively. For diadinoxanthin, EMS67 had 89.1% more accumulation than WT while EMS3 and EMS13 had 34.6% and 18.1% less accumulation than WT, respectively. For zeaxanthin, EMS3 had 17.4% more accumulation than WT while EMS7, EMS13, EMS30 and EMS67 had 14.8%, 12.5%, 23.1% and 22.7% more than WT, respectively. For chlorophyll *c*, EMS13 had 129.6% more accumulation than WT while EMS30 and EMS67 had 21.4% and 24.5% less accumulation than WT, respectively. As shown in Figure 4b, both EMS30 and EMS67 strains had higher chlorophyll *a* fluorescence intensity than WT. The Nile red fluorescence intensity of EMS30 was close to that of WT, but the Nile red fluorescence signal of EMS67 was much stronger than that of EMS30.

**Figure 5:**
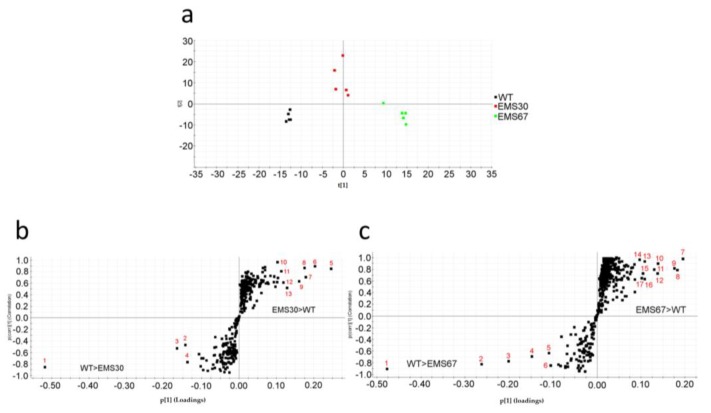
Phenotypic differentiation of WT and positive mutants. (**a**) PCA was used for sample grouping based on their metabolite profiles; (**b**) OPLS-DA S-plot showing the differences in production between WT and EMS30 groups: dots in the left lower quadrant are compounds contributing to the differentiation of WT from EMS30 with a potentially higher production in WT; dots in the right upper quadrant are compounds contributing to the differentiation of EMS30 from WT with a potentially higher production in EMS30; (**c**) OPLS-DA S-plot showing the differences in production between WT and EMS67 groups: dots in the left lower quadrant are compounds contributing to the differentiation of WT from EMS67 with a potentially higher production in WT, and dots in the right upper quadrant are compounds contributing to the differentiation of EMS67 from WT with a potentially higher production in EMS67.

With regard to neutral lipid content, all the strains had higher neutral lipid content than WT (Figure S4). For EMS7, EMS13, EMS30 and EMS67, the lipid content was 59.4%, 41.3%, 44.8% and 62.7% greater than that of WT, respectively. principal component analysis (PCA) was used to summarize the metabolite profiling data and cluster samples, including WT, EMS30 and EMS67 (Figure 5a). Higher intragroup variations were found in treated groups, while less variation was found in the WT group. The first component explained 25% of the chemical variation, mainly that between the WT and EMS67 groups, and the second component explained 18% of the variation, mainly that between the EMS30 and the WT groups. In OPLS-DA plots (Figure 5b,c), the horizontal axis indicated intergroup variation. OPLS-DA was performed well based on its goodness-of-fit parameter (R^2^ > 0.9) and predictive ability parameter (Q^2^ > 0.9). The vertical axis indicated the intragroup variation, and in both OPLS-DA plots, the WT group showed less variation than the treated groups. Markers identified as contributing to intergroup differentiation are labeled in the S-plots and reported in Supplementary Table S2 and most of the lipophilic markers identified were fatty acids. Both the PCA and OPLS-DA plots’ results confirmed phenotypic differences between WT and mutants. 

### 2.5. Assessment of Selected Mutant Stability for Carotenoid Accumulation 

**Figure 6:**
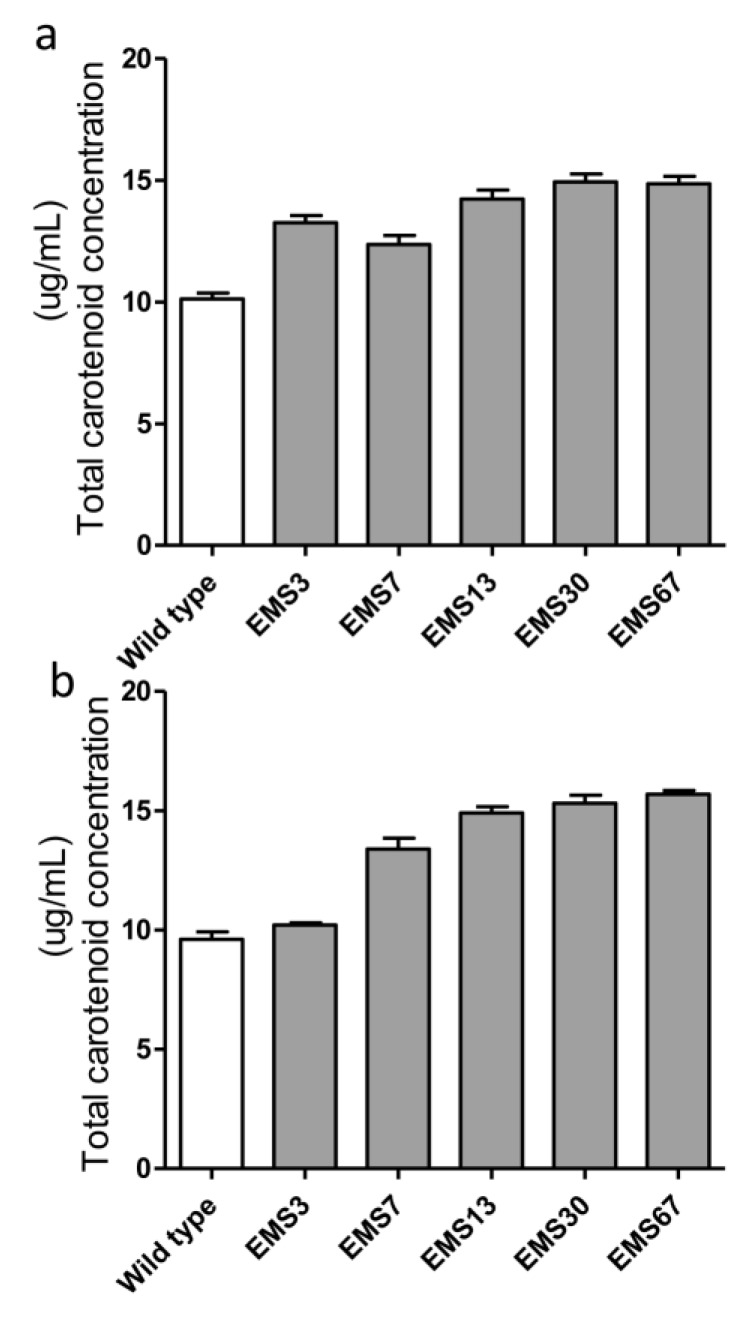
Stability evaluation of carotenoid accumulation in selected mutants. All strains were in the exponential growth phase, and the total carotenoids of mutants were measured both at the beginning of two months of Erlenmeyer flask cultivation (**a**) and at the end of two months of repeated batch culture in Erlenmeyer flasks (**b**). Each value was averaged from biological triplicates.

After our three-step selection (Figure 1), five positive mutants were chosen for stability analysis. The accumulation of total carotenoids in the selected strains before and after two months of repeated batch cultivation was also quantified. As shown in Figure 6a, before continuous cultivation, all five selected mutants had higher carotenoid content than the WT. EMS7 had the lowest content among the five mutants but had 22.5% more carotenoids than WT, while EMS30 and EMS67 had 47.4% and 46.7% more than WT, respectively. After two months of repeated batch cultivation (with approximately 16 generations had passed), four strains had almost identical carotenoid content as they did previously, with changes less than 10% (Figure 6b). Nevertheless, total carotenoid content in EMS3 dropped from 13.3 mg/g DW to 10.2 mg/g DW, nearly to the same level as WT. 

## 3. Discussion

To date, there are still gaps between diatom research development and its fully commercial applications [8,25]. It is essential to enhance the production of valuable compounds in diatoms towards commercialization. In this study, we utilized both EMS and NTG chemical mutagens to mutate *P. tricornutum* and designed an efficient screening process to select for desired phenotypes.

It has been reported that fatty acids and particular lipid compositions are closely linked with carotenoid accumulation in *Dunaliella salina* and *Haematococcus* sp*.* [22,26,27,28]. In addition, the correlation between Nile red fluorescence intensity and total carotenoid content was established in *D. salina* [22] with a coefficient of 0.74 in the exponential growth phase. The mechanism causing the correlation between lipid metabolism and carotenoid synthesis is yet to be explored, although studies have demonstrated that inhibition of carotenoid synthesis did not interfere with lipid metabolism [26,27,28]. Biochemical research shows that pyruvate is a precursor of both lipids and carotenoids and that pyruvate is converted to acetyl-CoA via the pyruvate dehydrogenase complex (PDC) in lipid metabolism or converted to 1-deoxy-d-xylulose 5-phosphate (DXP) via DXP synthase (DXS) [29]. In addition, carotenoids are lipophilic and synthesized in oil-rich chloroplasts; lipid globules also participate in carotenogenesis-related steps as transportation or modifications [26]. Although phytoene synthase and phytoene desaturase protein abundance and mRNA expression remained constant while beta-carotene was over-expressed under active lipid biosynthesis in *Dunaliella*, their enzymatic activities were significantly increased because the enzymatic activities were not necessarily related to protein and mRNA amount [26].

The intrinsic membrane antenna proteins in diatoms are fucoxanthin-chlorophyll-binding proteins (FCPs), which are located on thylakoids and serve both photosystems I and II [5]. The FCPs share homology with light-harvesting complexes (LHCs), but there are still large differences between them in terms of pigment composition and pigment ratio. The molar ratio of chlorophyll *a* to carotenoid in FCPs of diatoms is ~1, but the ratio of chlorophyll *a* to carotenoid is close to 2 in LHCs [6,30]. Diatom thylakoids were enlarged, and the expression of chlorophyll *a* was transcriptionally increased to fully utilize irradiated photons under low light conditions in exponential growth [5]. In this study, the molar ratio of chlorophyll *a* to carotenoids was relatively stable (varied from approximately 1.27 to 1.42) in the exponential growth phase in various mutants despite the large differences between carotenoid contents. This result implies that fucoxanthin metabolism may synergize with chlorophyll *a* accumulation to achieve appropriate ratios for optimal photosynthetic efficiency at utilizing luminous energy, while mutants that could not reach this ratio and failed to grow as quickly in colonies would not be selected for the following screening. As pigmentation and pigment composition are extremely sensitive to environmental conditions such as light intensity and quantity, pH, temperature, and nutrient availability [31], it is essential that the same growth conditions and collection times are strictly maintained for all strains and particularly for chlorophylls, as they are the most labile compounds. Chlorophyll *a* fluorescence could change dramatically as a result of external stimulants or internal growth phases [5], indicating that extreme attention should be paid to maintaining samples properly and measuring the fluorescence at identical designated times. Nevertheless, previous UV mutagenesis results [3] disclosed that most selected positive UV mutants had similar or lower beta-carotene and chlorophyll *a* content than WT, which was different that the situation found in the EMS positive mutants. Both beta-carotene and chlorophyll *a* had higher expression levels in EMS mutants than in WT (Figure 4a). The differences in UV and EMS mutants could be explained by their different mutagenesis mechanisms: UV promotes dipyrimidine sites forming cyclobutene pyrimidine dimers and pyrimidine-pyrimidone products that induce DNA damage [32], while EMS alkylates guanine, which induces mispairing alkylated G with T, causing G/C to A/T transitions [33]. 

The comprehensive genome-scale network reconstruction was structured on biochemical and genetic information from literature and has provided a scheme to study and evaluate the unexplored metabolic capabilities in diatoms [23]. To explore possible key enzymatic reactions that were involved in the correlations, we simulated the metabolism of *P. tricornutum* with the published iLB1025 model [23]. Randomized flux distributions within the model were estimated and then identified the enzymatic reactions linearly correlated with fucoxanthin production were then identified. We explored reactions across six compartments: cytosol, mitochondrion, extracellular space, chloroplast, peroxisome and thylakoid. Reactions in chlorophyll *a* and lipid metabolism correlated with flux in fucoxanthin production (Table S3). In porphyrin and chlorophyll metabolism, 13 reactions of a set of 25 reactions that we analyzed had linear correlations with fucoxanthin synthesis; 10 of these 13 were positively correlated, while the other three had negative correlations. Based on the Kyoto Encyclopedia of Genes and Genomes (KEGG) metabolic pathway maps, these 13 reactions belong to the chlorophyll *a* biosynthetic pathway. Among 439 total analyzed lipid metabolic reactions, 12 reactions that mostly belong to fatty acid elongation reactions were linearly correlated with fucoxanthin production. Partially correlated lipid and chlorophyll synthetic reactions were exhibited, and the highly correlated reactions are labeled in red (Figure S3). We also examined the correlated reactions in nucleotide metabolism, starch and sucrose metabolism, fructose and mannose metabolism and biosynthesis of steroids. The highly correlated reactions are also summarized (Table S3). In addition to the overlapping precursors, certain interactions between the translational or transcriptional regulation of these metabolites could also play an important role in the correlations. It would be intriguing to study the impacts of genetic or metabolic manipulations of these predicted highly correlated reactions on the production of fucoxanthin.

The metabolic pathways for synthesizing chlorophyll *a* and fucoxanthin share a few precursors, from glyceraldehyde 3-phosphate (GA3P) and pyruvate to geranylgeranyl pyrophosphate (GGPP). In fucoxanthin metabolism, GGPP is first converted to prephytoene-PP and then to phytoene under catalysis by phytoene synthase. For chlorophyll *a* metabolism, GGPP is converted to phytyl-PP by catalysis via geranylgeranyl reductase (GGDR), and then phytyl-PP is combined with chlorophyllide to synthesize chlorophyll *a* under chlorophyll synthase (CHLG) catalysis [34]. Lipid and fucoxanthin metabolism share the early precursors GA3P and pyruvate; DOXP synthase catalyzes the conversion of pyruvate and GA3P into DOXP in fucoxanthin metabolism, while pyruvate dehydrogenase converts pyruvate into acetyl-CoA in lipid metabolism [35].

As chlorophyll *a* and neutral lipid content could be determined spectrophotometrically in a high-throughput fashion, these findings enable the high-throughput screening of fucoxanthin-hyperproducing strains in diatoms by the development of fluorescence-based approaches for estimating fucoxanthin content. A comparison of Figure 4a and Figure 6b shows that the LC-MS data of fucoxanthin were consistent with the total carotenoids extracted with methanol. Four of 5 selected positive mutants showed stability in total carotenoid accumulation over 2 months repeated batch cultivation. The fading of fucoxanthin in one of the mutants may be because *P. tricornutum* is diploid [20,21] and the alleles for a particular gene are not mutated simultaneously. Therefore, it is suggested that the production stability of all selected mutants of *P. tricornutum* should be checked over long-term repeated cultivation.

The fluorescence-based high-throughput screening method developed here demonstrated efficiency advantages over conventional screening methods. In this study, five mutants were selected from approximately 1000 seeded mutated strains by fluorescence-based screening. This method combined fluorescence detection and agar plate and microplate cultivation, which enables the possibilities of large-scale mutagenesis screening, a key factor in creating prominent mutants. Different from one-by-one traditional spectrophotometer methods, the fluorescence detection that was established and based on a correlation between chlorophyll *a* fluorescence and total carotenoid content enabled an indirect and nondestructive approach to estimating fucoxanthin content in diatom cells. In general, this method could significantly increase the screening efficiency to obtain fucoxanthin-hyperproducing strains of diatoms. Furthermore, this screening method may be applied in other algal species that have a broad prospect in creating strains hyperproducing carotenoids. This high-throughput screening method may be attempted on any species whose carotenoid content has a satisfying correlation with chlorophyll *a* and/or neutral lipid content.

## 4. Material and Methods

### 4.1. Cells and Chemicals 

The *Phaeodactylum tricornutum* (CCAP 1055/1) strain was from the Culture Collection of Algae and Protozoa (CCAP), Scotland, the U.K. All of the chemicals were purchased from Sigma-Aldrich unless otherwise specified. Bidistilled water was generated using a Milli-Q System (Millipore, Bedford, MA, USA).

### 4.2. Diatom Culture and Growth

Diatoms were cultivated at 22.0 ± 2 °C in modified f/2 medium in which the pH was maintained at 8.0 ± 0.5. Cultures with a volume of 50 mL were grown in 250 mL Erlenmeyer flasks under continuous radiation with a light intensity of 30 μE/m^2^/s by daylight lamp (Osram, TEKNE, BL1, 73061-48, Munich, Germany) unless otherwise indicated. The light intensity was measured by a quantum sensor (Model LI-1400, LI-COR biosciences, Lincoln, NE, USA) to ensure persistent and steady illumination. The optical density at 625 nm (OD_625_) was used to determine the dry weight (DW) of the biomass [36,37]. The correlation of the biomass DW and OD_625_ was demonstrated (Figure S1). 

### 4.3. EMS and NTG Mutagenesis

For EMS mutagenesis*,* the *P. tricornutum* strain at a cell density of 1 × 10^6^ cells/ml was treated with either 0.1 M or 0.2 M EMS; for NTG mutagenesis, *P. tricornutum* was exposed to either 0.1 mM or 0.2 mM NTG. Both treatments were sustained for 1 h in dark at room temperature. After each treatment, *P. tricornutum* cells were washed thrice with 5% sodium thiosulfate to remove remaining mutagens, followed by being washed twice with fresh f/2 medium. The cells were kept in a dark room overnight to prevent light-reactivation and then seeded in f/2 agar plates under fluorescent lamp irradiation. After approximately 15 days of cultivation, single colonies with deep color and large sizes were selected for further cultivation. 

### 4.4. Herbicide Test

The herbicide DPA was dissolved in f/2 medium at different concentrations: 10 µM, 20 µM, 30 µM, 40 µM, 50 µM and 60 µM. *P. tricornutum* were seeded in 48-well plates at an initial density of 1 × 10^6^ cells/ml, and cells were illuminated with 30 μE/m^2^/s from a daylight lamp. After four days of cultivation, cells were collected and cell numbers were counted with a hemocytometer.

### 4.5. Chlorophyll a Fluorescence and Nile Red Staining Measurement

Both chlorophyll *a* and Nile red fluorescence were measured with a SpectraMax M3 Multi-mode Microplate Reader (Molecular Devices, Sunnyvale, CA, USA). For chlorophyll *a* fluorescence detection, the excitation wavelength was set at 440 nm, and emission was measured at a wavelength of 680 nm. Nile red is a high-affinity lipophilic dye that binds lipids and emits fluorescence under excitation. Its staining method was mentioned in a previous article.

### 4.6. Confocal Imaging 

For imaging, fluorescence was measured using an Olympus FV1200 Confocal microscope (Olympus, Tokyo, Japan). Differential interference contrast (DIC), chlorophyll *a* fluorescence, Nile red fluorescence images were acquired. Strains were in the exponential growth phase, and the settings for the observation for each strain were identical: the excitation laser wavelength for chlorophyll *a* and Nile red was 488 nm, and the optical emission filter allowed light between 560 nm and 620 nm for Nile red and between 655 nm and 750 nm for chlorophyll *a*.

### 4.7. Spectrophotometer for Pigment Detection

Spectrophotometric method was applied to estimate chlorophyll *a* and total carotenoid content based on pigment extraction [38]. Samples were transferred to a 1 mL EP tube and centrifuged at 10,000 RPM for 20 min, and then supernatants were discarded. Next, 1 mL 100% (*v*/*v*) methanol was added to each tube, whose contents were then vigorously pipetted and vortexed. Samples were sonicated for 1 h and centrifuged again, and supernatants were collected. The optical absorbances at 665 nm, 652 nm and 470 nm were measured [38]. The equations for chlorophyll *a* content and total carotenoid content are below:

Ca = 15.65 × A_665_ − 7.34 × A_652_, (1)

Xcarotenoids = ((1000 × A_470_) − (2.86 × Ca))/221. (2)

Ca represents chlorophyll *a* content, Xcarotenoids represents total carotenoid content and A_665_, A_652_, and A_470_ represent the optical absorbance at each wavelength, respectively.

### 4.8. LC-MS Determination and Analysis of Major Pigments

As described in former studies [39], we also used an ACQUITY UPLC coupled to a SYNAPT G2 HDMS system (Waters, Milford, MA, USA), which was equipped with an HSS T3 1.8 μm column (2.1 × 150 mm; Waters, Manchester, United Kingdom). The same applies to flow rates and the used gradient and mobile phases, respectively (Phase A: ACN:MetOH:MTBE = 70:20:10 (*v*:*v*:*v*); Phase B: water with 10mM Ammoniumacetate). 

Ten concentration steps (1.7–400mg/L) of standards of β-carotene (CAS-no.: 7235-40-7), fucoxanthine (CAS-no.: 3351-86-8) and chlorophyll *a* (CAS-no.: 479-61-8) were created by diluting pure substances in pure isopropanol in order to calibrate those three substances as well as to validate the accuracy of our detection according to the formerly described methods [39]. In the beginning of daily LC-MS-batches, 10 measurements of pooled samples were performed to equilibrate the column. Through this, we were given proof that retention time shifts and decreased sensitivity have not occurred within daily batches. Between measurements’ runs of pure isopropanol were performed to prevent carry-over-effects. Identifications of carotenoids other than the calibrated ones were realized using *m*/*z*-ratios and retention times formerly described in studies using the same or similar LC-MS-methods [3,39].

### 4.9. Metabolic Modeling Analysis

The *i*LB1025 genome-scale reconstructed metabolic model of *P. tricornutum* was applied to predict metabolic reactions correlated with fucoxanthin production [23]. The model was analyzed by randomly sampling fluxes from the system. Random sampling generated numerous flux vectors on behalf of the system’s feasible states where single flux vector elements amount to the fluxes in individual reactions [40]. The correlation coefficients between fluxes in fucoxanthin production and other metabolic reactions in the system were calculated for the selection of the most correlated metabolic reactions. The CosMos algorithm was used to identify the correlation, and the computational analysis was executed in the MATLAB (9.1, The MathWorks, Natick, MA, USA) environment applying the COBRA toolbox version 2.0 [23,40].

### 4.10. Data Processing and Analysis

MassLynx v4.1 (Waters Corp., Milford, MA, USA) was used to identify and quantify cellular compounds. Principal component analysis (PCA) and OPLS-DA (orthogonal partial least squares discriminate analysis) were conducted by software SIMCA 14 (Sartorius Stedim, Malmö, Sweden). PCA was used to summarize the metabolite profiling data and reveal the grouping of samples. OPLS-DA was used for group–group comparisons, and OPLS-DA S-plots were applied to visualize the metabolites that contribute most significantly to the intergroup variations [41]. Before applying the PCA, data were normalized through summing, log transformation, and scaled to a mean of zero and unit standard deviation. Intergroup comparison was applied by one-way ANOVA.

### 4.11. High-Throughput Screening Method

The high-throughput screening method was comprised of three steps of screening. After chemical mutagenesis, mutants were cultivated evenly in f/2 agar plates with 40 µM DPA. After 15 days under 30 μE/m^2^/s continuous illumination, colonies with darker color and larger size were selected and cultured in f/2 medium in microplates. Strains were re-seeded every 7 days, and the initial biomass density was kept at approximately 0.06 (OD_625_). For the secondary screening, 200 μL medium of each strain was transferred to 96-well microplates in triplicate after 36 h cultivation of each initial seeding to keep strains in identical exponential growth phases. The optical density, chlorophyll *a* fluorescence intensity and Nile red fluorescence values were averaged from triplicate measurements. The equations we utilized are listed below: RFU_chlo*a*_ represents the relative fluorescence intensity of chlorophyll *a*, and RFUnr represents the Nile red fluorescence intensity:

RFU_chlo*a*_ ≥ 2309 × OD_625_ − 24.3, (3)

RFUnr ≥ 167.1 × OD_625_ − 0.21. (4)

Strains screened via Equations (3) and (4) were picked out and cultivated in 48-well plates for the next screening step. Total carotenoids and chlorophyll *a* contents, which had been extracted with 100% methanol, were calculated based on previously described equations [38]. Positive mutants whose total carotenoids were at least 15% higher than those of WT were selected for further cultivation in Erlenmeyer flasks. After two months of repeated batch cultivation, pigment content was re-examined, followed by methanol extraction to evaluate the strain stability. Specific pigment content of strains grown in Erlenmeyer flasks was determined through LC-MS. The schematic process is demonstrated in Figure 1.

## Supplementary Materials

The following are available online at http://www.mdpi.com/1660-3397/16/8/272/s1: Supplementary Figure S1: Correlation between optical density at 625 nm and diatom dry biomass concentration; Supplementary Figure S2: Effects of DPA concentration on *P. triconrnutum* growth; Supplementary Figure S3: Intersectional metabolic pathways for carotenogenesis, chlorophyll *a* production and lipid metabolism; Supplementary Figure S4: Relative Nile red fluorescence intensity for selected strains; Supplementary Table S1: Equations for the correlations of total carotenoids with chlorophyll *a* or lipids content; Supplementary Table S2: Enzymatic reactions with the highest correlation with fluxes in the fucoxanthin production. The data was obtained through conducting various flux analysis in published iLB1025 model; Supplementary Table S3: Tentative identification of different lipophilic compound expression in both WT and mutants.
