# Supplementary material for: Chemical Mutagenesis and Fluorescence-Based High-Throughput Screening for Enhanced Accumulation of Carotenoids in a Model Marine Diatom Phaeodactylum tricornutum
Source: Mar Drugs. 2018 Aug 4;16(8):272. doi: 10.3390/md16080272 (PMC6117690; doi:10.3390/md16080272)
Supplement: Supplementary file 1 [file marinedrugs-16-00272-s001.zip › Supplementary Figures.pdf]

SUPPLEMENTARY FIGURES

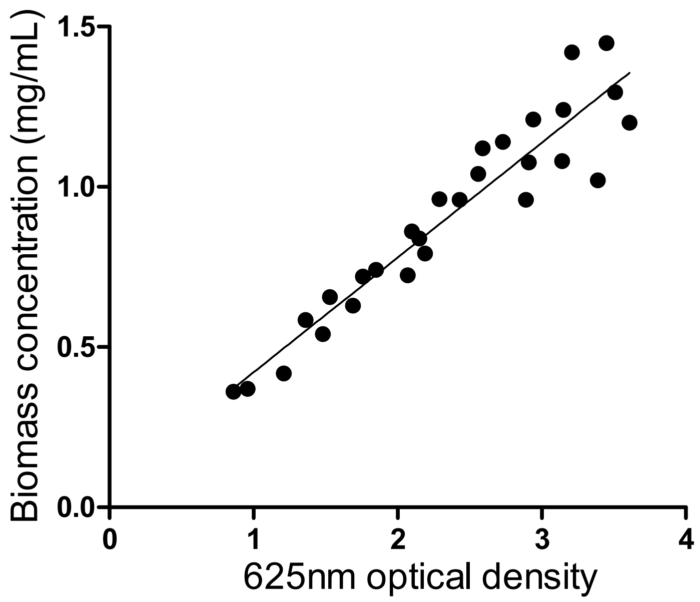

Supplementary Figure 1. Correlation between optical density at 625 nm and diatom dry biomass concentration. Diatom cell samples were harvested on a cellulose membrane (pore size, 0.45 μm) and washed twice with deionized water dried at 60 °C overnight, dried at 60 °C overnight.

p

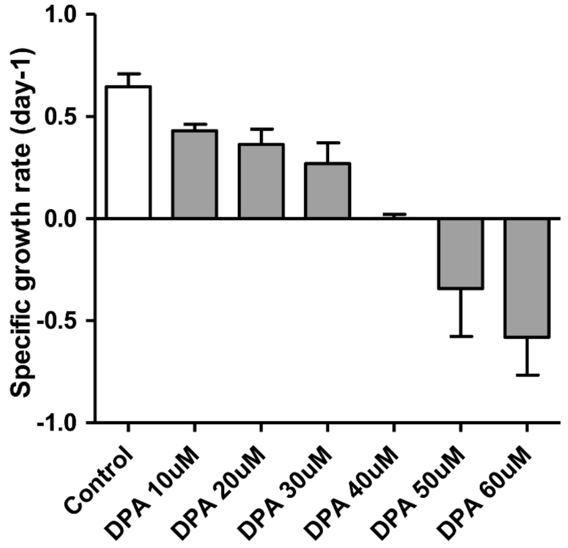

Supplementary Figure 2. Effects of DPA concentration on P. triconrnutum growth. P. tricornutum were incubated with different concentrations of DPA: 10 μM, 20 μM, 30 μM, 40 μM, 50 μM, and 60 μM for 4 days, respectively. Specific growth rate (μ) is defined as the rate of increase of biomass of a cell population per unit of biomass concentration and was measured in biological triplicates for each treatment.

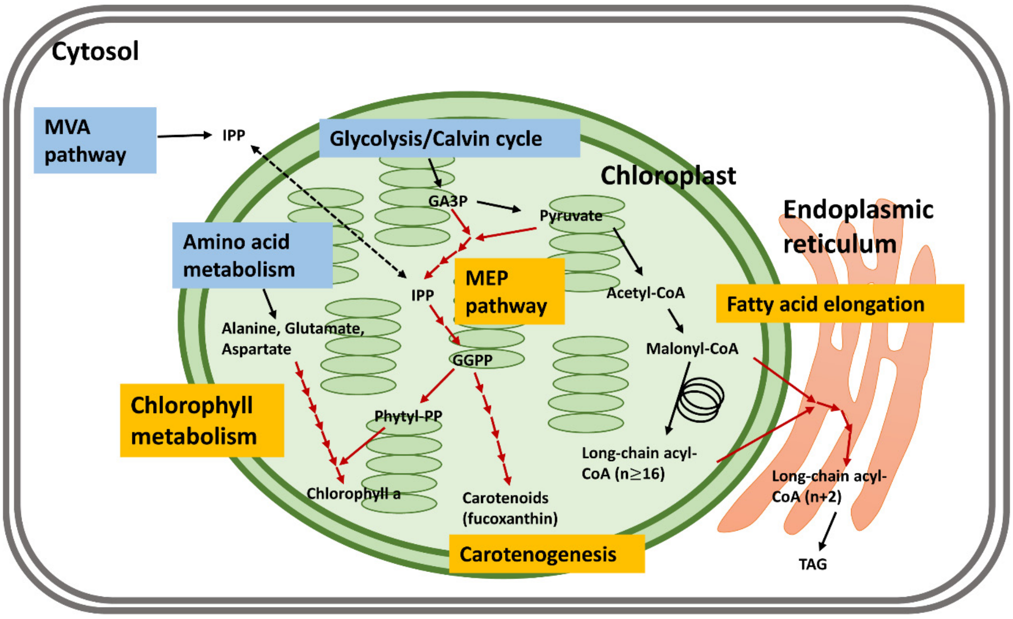

Supplementary Figure 3. Intersectional metabolic pathways for carotenogenesis, chlorophyll *a* production and lipid metabolism.

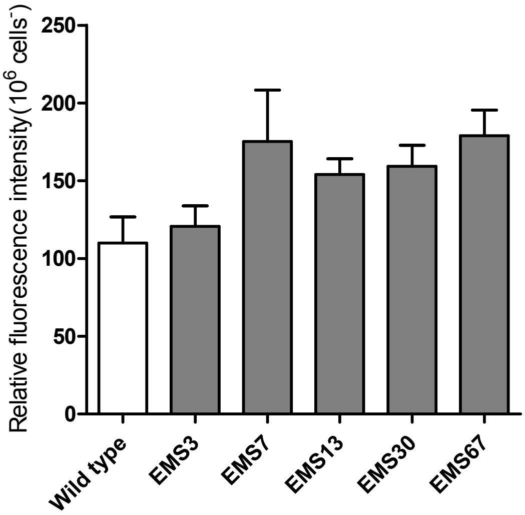

Supplementary Figure 4. Relative Nile red fluorescence intensity for selected strains. Strains were incubated with 1ug/mL Nile red for 20min in dark room. Each fluorescence value was averaged from triplicates.
