# Supplementary material for: Chemical Mutagenesis and Fluorescence-Based High-Throughput Screening for Enhanced Accumulation of Carotenoids in a Model Marine Diatom Phaeodactylum tricornutum
Source: Mar Drugs. 2018 Aug 4;16(8):272. doi: 10.3390/md16080272 (PMC6117690; doi:10.3390/md16080272)
Supplement: Supplementary file 1 [file marinedrugs-16-00272-s001.zip › Supplementary Table 1.pdf]

	Equations
a)	y=0.0025x+0.0123 (R^2=0.8687)<br>(x: relative chlorophyll *a* fluorescence intensity; y: total carotenoid content)
b)	y=0.0021x+0.1501 (R^2=0.7395)<br>(x: relative chlorophyll *a* fluorescence intensity; y: total carotenoid content)
c)	y=0.018x-0.3253 (R^2=0.6356)<br>(x: relative Nile red fluorescence intensity; y: total carotenoid content)
d)	y=0.0081x+0.8609 (R^2=0.3758)<br>(x: relative Nile red fluorescence intensity; y: total carotenoid content)

Supplementary Table 1: Equations for the correlations of total carotenoids with chlorophyll a or lipids content (indicated by Nile red fluorescence intensity).
