# Supplementary material for: Chemical Mutagenesis and Fluorescence-Based High-Throughput Screening for Enhanced Accumulation of Carotenoids in a Model Marine Diatom Phaeodactylum tricornutum
Source: Mar Drugs. 2018 Aug 4;16(8):272. doi: 10.3390/md16080272 (PMC6117690; doi:10.3390/md16080272)
Supplement: Supplementary file 1 [file marinedrugs-16-00272-s001.zip › Supplementary Table 2.pdf]

Table S2. Enzymatic reactions with the highest correlation with fluxes in the fucoxanthin production. The data was obtained through conducting various flux analysis in published iLB1025 model.

Reaction	Correlation	KEGG ID	EC	Catalyst
**Nucleotide metabolism: Pyrimidine metabolism**				
TDSR_c	1.00	R02016	1.8.1.9	Thioredoxin-disulfide reductase
OMPDC_c	1.00	R00965	4.1.1.23	Orotidine-5"-phosphate decarboxylase
MDUMT_c	1.00	R02101	2.1.1.45	5,10-Methylenetetrahydrofolate:dUMP C-methyltransferase
ASPCT_c	1.00	R01397	2.1.3.2	Aspartate carbamoyltransferase
DHR_c	-1.00	R01993	3.5.2.3	Dihydroorotase
ORPRT_c	-1.00	R01870	2.4.2.10	Orotate phosphoribosyltransferase
DHRDH_c	1.00	R01867	1.3.98.1	Dihydroorotate dehydrogenase (Fumarate)
UTAL_c	0.98	R00571	6.3.4.2	UTP:ammonia ligase (ADP-forming)
DCDT_c	0.81	R02024	1.17.4.1	2'-Deoxycytidine diphosphate:oxidized-thioredoxin 2'-oxidoreductase
ATDCM_c	-0.61	R01665	2.7.4.14	ATP:dCMP phosphotransferase
DCMAH_c	0.61	R01663	3.5.4.12	dCMP aminohydrolase
ITCY_c	0.26	R00962	2.7.1.48	ITP:cytidine 5"-phosphotransferase
CMP_c	0.26	R00511	3.1.3.5	Cytidine-5"-monophosphate phosphohydrolase
DURIPP_c	0.15	R02484	2.4.2.1	Deoxyuridine phosphorylase
UPRT_c	-0.15	R00966	2.4.2.9	Uracil phosphoribosyltransferase
NTD1_c	0.15	R02102	3.1.3.5	2"-Deoxyuridine 5"-monophosphate phosphohydrolase
**Nucleotide metabolism: Purine metabolism**				
DGOTO_c	0.64	R02019	1.17.4.1	2'-Deoxyguanosine 5'-diphosphate:oxidized-thioredoxin 2'-oxidoreductase

DAOTO_c	0.56	R02017	1.17.4.1	2'-Deoxyadenosine 5'-diphosphate:oxidized-thioredoxin 2'-oxidoreductase
AIAL_c	0.21	R04559	4.3.2.2	1-(5"-Phosphoribosyl)-5-amino-4-(N-succinocarboxamide)-imidazole AMP-lyase
PRAIS_c	0.21	R04591	6.3.2.6	Phosphoribosylaminoimidazolesuccinocarboxamide synthase
PRAIC_c	-0.21	R04209	4.1.1.21	Phosphoribosylaminoimidazole carboxylase
ITPA_c	0.20	R00719	3.6.1.5	ITP-apyrase
DAMPH_c	-0.17	R02088	3.1.3.5	2"-Deoxyadenosine 5"-monophosphate phosphohydrolase
ATDAM_c	0.17	R01547	2.7.4.3	ATP:dAMP phosphotransferase
FPGFT_c	0.16	R04325	2.1.2.2	Phosphoribosylglycinamide formyltransferase
PPRGL_c	0.16	R04144	6.3.4.13	Phosphoribosylamine-glycine ligase
PRFGS_c	0.16	R04463	6.3.5.3	Phosphoribosylformylglycinamidine synthase
PRDPAR_c	0.16	R01072	2.4.2.14	5-Phosphoribosylamine:diphosphate phospho-alpha-D-ribosyltransferase
PRFGCL_c	0.16	R04208	6.3.3.1	Phosphoribosylformylglycinamidine cyclo-ligase
GPAR_c	0.15	R01229	2.4.2.7	GMP:diphosphate 5-phospho-alpha-D-ribosyltransferase
PUNP3_c	0.15	R02147	2.4.2.1	Purine-nucleoside phosphorylase (Guanosine)
GMP5N	0.15	R01227	3.1.3.5	GMP-5"-nucleotidase
XPPRT_c	-0.14	R02142	2.4.2.8	XMP:pyrophosphate phosphoribosyltransferase
PUNP7_c	-0.14	R02297	2.4.2.1	Purine-nucleoside phosphorylase (Xanthosine)
X5NT_c	-0.14	R02719	3.1.3.5	XMP-5"-nucleotidase
**Nitrogen metabolism**				

NITR_c	0.86	R00794	1.7.1.1	Nitrate reductase (NADH)
NTRIR_h	0.34	R02016	1.8.1.9	Nitrite reductase (NADPH), chloroplast
NOR_c	0.25	R00790	1.7.7.1	Ferredoxin-Nitrite Reductase
**Nucleotide sugar metabolism**				
XYLE_c	1.00	R01473	5.1.3.5	UDP-xylose 4-epimerase
UDPRHMS_c	1.00			UDP-beta-L-rhamnose synthase
GDPMANST_c	1.00			GDP-mannose-3-sulftransferase
UDPGLDC_c	1.00			UDP-D-glucuronate decarboxylase
**Starch and sucrose metabolism**				
UGDH	1.00	R00286	1.1.1.22	UDP-glucose 6-dehydrogenase
**Fructose and mannose metabolism**				
GFUCS_c	1.00	R05692	1.1.1.271	GDP-L-fucose synthase
GMAND_c	1.00	R00888	4.2.1.47	GDP-D-mannose dehydratase
**Biosynthesis of steroids: Terpenoid backbone synthesis**				
GPPS_h	1.00	R01658	2.5.1.1	geranyl pyrophosphate synthase, chloroplast
CMK_h	1.00	R05634	2.7.1.148	4-(cytidine 5'-diphospho)-2-C-methyl-D-erythritol kinase
GGPS_h	1.00	R02061	2.5.1.29	Geranylgeranyl diphosphate synthase
DXR_h	1.00	R05688	1.1.1.267	1-deoxy-D-xylulose-5-phosphate reductoisomerase
MECDPS_h	1.00	R05637	4.6.1.12	2-C-methyl-D-erythritol 2,4-cyclodiphosphate synthase
GGDR_h	1.00	R02063	1.3.1.83	geranylgeranyl diphosphate reductase

HMBDPO_h	1.00	R08689	1.17.7.1	4-hydroxy-3-methylbut-2-en-1-yl-diphosphate:oxidized ferredoxin oxidoreductase
CMS_h	1.00	R05633	2.7.7.60	2-C-methyl-D-erythritol 4-phosphate cytidylyltransferase
FPPS_h	1.00	R02003	2.5.1.10	Farnesyl pyrophosphate synthase
DXPS_h	0.73	R05636	2.2.1.7	1-deoxy-D-xylulose 5-phosphate synthase
IDS1_h	0.55	R05884	1.17.1.2	isopentenyl-diphosphate synthase
IDIH_h	0.20	R01123	5.3.3.2	isopentenyl-diphosphate Delta-isomerase, chloroplast
**Carotenoid biosynthesis**				
NOR_h	-1.00	R04800	1.3.5.6	Neurosporene oxidoreductase
PSY_h	1.00	R02065<br>R07270	2.5.1.32	phytoene synthase
ZDS_h	-1.00	R04798	1.3.5.6	zeta-carotene desaturase
BCAROXR_h	1.00	R07558<br>R07559		Beta-carotene, NADH:oxygen 3-oxidoreductase (zeaxanthin forming)
NSY_h	-1.00	R06948	5.3.99.9	Neoxanthin synthase
FXANS_h	1.00			Fucoxanthin synthase
PDS1_c	1.00	R04786	1.3.5.5	phytoene desaturase (1)
PDS2_h	1.00	R04787	1.3.5.5	phytoene desaturase (2)
LYCBC1_c	-1.00	R05341	5.5.1.19	Lycopene cyclase (gamma-carotene producing)
LYCBC2_h	1.00	R03824	5.5.1.19	Lycopene cyclase (beta-carotene producing)
DIADINX_h	1.00			Diadinoxanthin synthase
**Porphyrin and chlorophyll metabolism**				

PPBNGD_h	-1.00	R00084	2.5.1.61	porphobilinogen deaminase
UPPS_h	1.00	R03165	4.2.1.75	Uroporphyrinogen-III synthase
MPOXR_h	1.00	R10068	1.14.13.81	Magnesium-protoporphyrin-IX 13-monomethyl ester,NADPH:oxygen oxidoreductase
PPBNGS_h	1.00	R00036	4.2.1.24	porphobilinogen synthase
PPPGO_h	1.00	R03222	1.3.3.4	protoporphyrinogen IX oxidase
CHLPAS_h	1.00	R06284	2.5.1.62	Chlorophyll A synthase
CPPPGO_h	1.00	R03220	1.3.3.3	coproporphyrinogen III oxidase (O2 required)
CHLPC1S_h	1.00			Chlorophyll C1 synthase
G1SAT_h	-1.00	R02272	5.4.3.8	glutamate-1-semialdehyde aminotransferase
GLUTRR_h	1.00	R04109	1.2.1.70	Glutamyl-tRNA reductase
CHLPC2S_h	1.00			Chlorophyll C2 synthase
MPML_h	1.00	R03877	6.6.1.1	Mg-protoporphyrin IX magnesium-lyase
MPMT_h	-1.00	R04237	2.1.1.11	S-adenosyl-L-methionine:Mg-protoporphyrin IX methyltransferase
DVPCHLDR_h	0.56	R06896	1.3.1.75	Divinylprotochlorophyllide vinyl-reductase
PCHLDOR_h	0.54	R03845	1.3.1.33	Protochlorophyllide oxidoreductase (light-dependent)
UPP3DC_h	0.19	R03197	4.1.1.37	uroporphyrinogen decarboxylase (uroporphyrinogen III)
UPP3DC_c	0.16	R03197	4.1.1.37	Uroporphyrinogen decarboxylase (uroporphyrinogen III)
**Fatty acid biosynthesis**				
3OAS200_c	1.00	R09419	2.3.1.199	very-long-chain 3-oxoacyl-CoA synthase (20:0 forming)
3OAS220_c	1.00	R09419	2.3.1.199	very-long-chain 3-oxoacyl-CoA synthase (22:0 forming)
3OAS240_c	1.00	R09419	2.3.1.199	very-long-chain 3-oxoacyl-CoA synthase (24:0 forming)
3OAR200_c	1.00	R01779	1.1.1.330	very-long-chain 3-oxoacyl-CoA reductase (20:0 forming)

3OAR220_c	1.00	R01779	1.1.1.330	very-long-chain 3-oxoacyl-CoA reductase (22:0 forming)
3OAR240_c	1.00	R01779	1.1.1.330	very-long-chain 3-oxoacyl-CoA reductase (24:0 forming)
3HAD200_c	1.00	R02685	4.2.1.134	very-long-chain (3R)-3-hydroxyacyl-CoA dehydratase (20:0 forming)
3HAD220_c	1.00	R02685	4.2.1.134	very-long-chain (3R)-3-hydroxyacyl-CoA dehydratase (22:0 forming)
3HAD240_c	1.00	R02685	4.2.1.134	very-long-chain (3R)-3-hydroxyacyl-CoA dehydratase (24:0 forming)
EAR200_c	1.00	R09449	1.3.1.93	very-long-chain enoyl-CoA reductase (20:0 forming)
EAR220_c	1.00	R09449	1.3.1.93	very-long-chain enoyl-CoA reductase (22:0 forming)
EAR240_c	1.00	R09449	1.3.1.93	very-long-chain enoyl-CoA reductase (24:0 forming)
**Amino acid metabolism: aminoacyl-tRNA biosynthesis**				
CYSTL_c	1.00	R03650	6.1.1.16	Cysteine-tRNA ligase
ALATL_c	1.00	R03038	6.1.1.7	Alanine-tRNA ligase
LEUTL_c	1.00	R03657	6.1.1.4	Leucine-tRNA ligase
METTL_c	1.00	R03659	6.1.1.10	Methionine-tRNA ligase
TRPTL_c	1.00	R03664	6.1.1.2	Tryptophane-tRNA ligase
SERTL_c	1.00	R03662	6.1.1.11	Serine-tRNA ligase
GLUTL_h	1.00	R05578	6.1.1.17	Glutamate-tRNA ligase
GLUTL_c	1.00	R05578	6.1.1.17	Glutamate-tRNA ligase
ARGTL_c	1.00	R03646	6.1.1.19	Arginine-tRNA ligase
TYRTL_c	1.00	R02918	6.1.1.1	Tyrosine-tRNA ligase
ASNTL_c	1.00	R03648	6.1.1.22	Asparagine-tRNA ligase
LYSTL_c	1.00	R03658	6.1.1.6	Lysine-tRNA ligase
GLNTL_c	1.00	R03652	6.1.1.18	Glutamine-tRNA ligase
ASPTL_c	1.00	R05577	6.1.1.12	Aspartate-tRNA ligase

PHETL_c	1.00	R03660	6.1.1.20	Phenylalanine-tRNA ligase
THRTL_c	1.00	R03663	6.1.1.3	Threonine-tRNA ligase
GLYTL_c	1.00	R03654	6.1.1.14	Glycine-tRNA ligase
SERTL_h	-0.16	R03662	6.1.1.11	Serine-tRNA ligase, chloroplast
**Amino acid metabolism: Cysteine and Methionine metabolism**				
MTRK_c	1.00	R04143	2.7.1.100	S-methyl-5-thioribose kinase
DKPPHL_c	1.00	R07395	3.1.3.77	2,3-Diketo-5-methylthiopentyl-1-phosphate phosphohydrolase
MTRI_c	1.00	R04420	5.3.1.23	S-methyl-5-thioribose-1-phosphate isomerase
MTAN_m	1.00	R01401	3.2.2.9 3.2.2.16	methylthioadenosine nucleosidase - adenosylhomocysteine nucleosidase
DH5MTPOXR_c	1.00	R07364	1.13.11.54	1,2-dihydroxy-5-(methylthio)pent-1-en-3-one:oxygen oxidoreductase
MDRPD_c	1.00	R07392	4.2.1.109	5-Methylthio-5-deoxy-D-ribulose 1-phosphate dehydratase
TAL_m	0.21	R00996	4.3.1.19	threonine ammonia-lyase
THRS_c	0.20	R01466	4.2.3.1	threonine synthase
G3PAT140_h	1.00	R09380	2.3.1.15	glycerol-3-phosphate: acyl-ACP acyltransferase (14:0)
AGPATACP_PALM_PALM_h	1.00	R02241	2.3.1.51	1-Hexadecanoyl-sn-glycerol-3-phosphate O-acyltransferase (16:0) (ACP Substrate)
PAPA_EPA_PALM_h	1.00	R02239	3.1.3.4	Phosphatidate phosphatase(20:5(5Z,8Z,11Z,14Z,17Z)/16:0) chloroplast
CDPDAGS_EPA_PALM_h	1.00	R01799	2.7.7.41	CDP-diacylglycerol synthase (20:5(5Z,8Z,11Z,14Z,17Z)/16:0) chloroplast
G3PAT160_h	1.00	R09380	2.3.1.15	glycerol-3-phosphate: acyl-ACP acyltransferase (16:0)
AGPATACP_MYRS_PALM_h	1.00	R02241	2.3.1.51	1-Tetradecanoyl-sn-glycerol-3-phosphate O-acyltransferase (16:0) (ACP Substrate)

AGPATACP_EPA_PALM_h	1.00	R02241	2.3.1.51	1-5,8,11,14,17-Eicosapentaenoyl-sn-glycerol-3-phosphate O-acyltransferase (16:0) (ACP Substrate)
PAPA_MYRS_PALM_h	1.00	R02239	3.1.3.4	Phosphatidate phosphatase(14:0/16:0) chloroplast
SQDGS_MYRS_PALM_h	1.00			Sulfoquinovosyldiacylglycerol synthase(14:0/16:0)
SQDGS_EPA_PALM_h	1.00			Sulfoquinovosyldiacylglycerol synthase(20:5(5Z,8Z,11Z,14Z,17Z)/16:0)
SQDGS_PALM_PALM_h	1.00			Sulfoquinovosyldiacylglycerol synthase(16:0/16:0)
SQDGS_HDE_TTC_c	1.00			Sulfoquinovosyldiacylglycerol synthase(16:1(9Z)/24:0)
ASQ_EPA_PALM_2OEPA_h	1.00			Sulfoquinovosyl diacylglycerol-2-O-acyl transferase (20:5(5Z,8Z,11Z,14Z,17Z)/16:0)(SQDG-2-O-20:5(5Z,8Z,11Z,14Z,17Z))
**Phosphoglycerolipid metabolism**				
PGPP_EPA_PALM_h	1.00	R02029	3.1.3.27	Phosphatidylglycerol phosphate phosphatase (20:5(5Z,8Z,11Z,14Z,17Z)/16:0) chloroplast
PGD3TDS_EPA_HDE3T_h	1.00			Phosphatidylglycerol sn-2 palmitoyl delta 3 desaturase (20:5(5Z,8Z,11Z,14Z,17Z)/16:1(3E)) chloroplast
AGPATCOA_HDE_TTC_c	1.00	R02241	2.3.1.51	1-9-hexadecenoyl-sn-glycerol-3-phosphate O-acyltransferase (24:0) (CoA Substrate)
PAPA_HDE_TTC_c	1.00	R02239	3.1.3.4	Phosphatidate phosphatase (16:1(9Z)/24:0)
PGPS_EPA_PALM_h	1.00	R01801	2.7.8.5	Phosphatidylglycerol phosphate synthetase (20:5(5Z,8Z,11Z,14Z,17Z)/16:0) chloroplast
PAPA_PALM_PALM_h	1.00	R02239	3.1.3.4	Phosphatidate phosphatase (16:0/16:0)
UDPSQS_h	1.00	R05775	3.13.1.1	UDP-sulfoquinovose synthase

CDIPT_HDE_PALM_c	0.18	R01802	2.7.8.11	CDP-diacylglycerol: myo-inositol 3-phosphatidyl transferase (16:1(9Z)/16:0)
CDPDAGS_HDE_PALM_c	0.18	R01799	2.7.7.41	CDP-diacylglycerol synthase(16:1(9Z)/16:0)
DAGK_HDE_PALM_c	0.14	R02240	2.7.1.107	Diacylglycerol kinase(16:1(9Z)/16:0)
ACPT_EPA_EPA_c	1.00			Betaine lipid synthase (20:5(5Z,8Z,11Z,14Z,17Z)/20:5(5Z,8Z,11Z,14Z,17Z))
BPNT_c	1.00	R00188	3.1.3.7	3",5"-Bisphosphate nucleotidase (pap)
