# Supplementary material for: Chemical Mutagenesis and Fluorescence-Based High-Throughput Screening for Enhanced Accumulation of Carotenoids in a Model Marine Diatom Phaeodactylum tricornutum
Source: Mar Drugs. 2018 Aug 4;16(8):272. doi: 10.3390/md16080272 (PMC6117690; doi:10.3390/md16080272)
Supplement: Supplementary file 1 [file marinedrugs-16-00272-s001.zip › Supplementary Table 3.pdf]

**Supplementary Table 3.** Tentative identification of different lipophilic compound expression in both WT and mutants.

a) Lipophilic compounds' differences between WT and EMS30;

| Marker No. | Group | Supposed metabolite | RT (min) | m/z | formula | adduct | ppm |
|---|---|---|---|---|---|---|---|
| 1 | WT | eicosasphinganine | 9.54 | 312.3287 | C20H43NO2 | [M+H-H2O]+ | 6 |
| 2 | WT | Octadecanoic acid | 7.8 | 284.2979 | C18H39NO2 | [M+H-H2O]+ | 9 |
| 3 | WT | Docosanamide | 11.03 | 340.3603 | C22H45NO | [M+H]+ | 8 |
| 4 | WT | DGCC(16:0/20:5) | 10.82 | 756.5796 | C46H79NO8 | [M+H-H2O]+ | 2 |
| 5 | EMS30 | Antheraxanthin | 13.47 | 607.3956 | C40H56O3 | [M+Na]+ | 28 |
| 6 | EMS30 | PA(P-16:0/20:4) | 13.48 | 663.4583 | C39H69O7P | [M+H-H2O]+ | 24 |
| 7 | EMS30 | Ganoderic acid V | 13.48 | 551.3312 | C32H48O6 | [M+Na]+ | 5 |
| 8 | EMS30 | Hoslundin | 14.74 | 429.0912 | C23H18O7 | [M+Na]+ | 8 |
| 9 | EMS30 | PG(22:6/21:0) | 15.82 | 887.5728 | C49H85O10P | [M+Na]+ | 5 |
| 10 | EMS30 | Spectinomycin adenylate | 14.73 | 684.205 | C24H36N7O13P | [M+Na]+ | 7 |
| 11 | EMS30 | 4,4'-Sulfonylbisacetanilide | 14.72 | 355.0719 | C16H16N2O4S | [M+Na]+ | 1 |
| 12 | EMS30 | Benzyl Dimethylphosphonoacetate | 12 | 281.0555 | C11H15O5P | [M+Na]+ | 2 |
| 13 | EMS30 | PG(22:6/21:0) | 15.49 | 887.5737 | C49H85O10P | [M+Na]+ | 5 |

b) Lipophilic compounds' differences between WT and EMS67;

| Marker No. | Group | Supposed metabolite | RT (min) | m/z | formula | adduct | ppm |
|---|---|---|---|---|---|---|---|
| 1 | WT | eicosasphinganine | 9.54 | 312.3287 | C20H43NO2 | [M+H-H2O]+ | 6 |
| 2 | WT | Docosanamide | 11.03 | 340.3603 | C22H45NO | [M+H]+ | 8 |
| 3 | WT | N-Docosylacetamide | 12.24 | 368.3927 | C24H49NO | [M+H]+ | 11 |
| 4 | WT | Ixocarpanolide | 13.48 | 495.2662 | C28H40O6 | [M+Na]+ | 11 |
| 5 | WT | 4,4'-Sulfonylbisacetanilide | 15.27 | 355.0704 | C16H16N2O4S | [M+Na]+ | 1 |
| 6 | WT | Phe Cys Gly Leu | 13.5 | 439.2021 | C20H30N4O5S | [M+H]+ | 7 |
| 7 | EMS67 | Hoslundin | 14.74 | 429.0921 | C23H18O7 | [M+Na]+ | 8 |
| 8 | EMS67 | PG(22:6/21:0) | 15.82 | 887.5728 | C49H85O10P | [M+Na]+ | 5 |
| 9 | EMS67 | PG(22:6/21:0) | 15.49 | 887.5737 | C49H85O10P | [M+Na]+ | 5 |
| 10 | EMS67 | PA(P-16:0/20:4) | 13.48 | 663.4583 | C39H69O7P | [M+H-H2O]+ | 24 |
| 11 | EMS67 | 4,4'-Sulfonylbisacetanilide | 14.72 | 355.0719 | C16H16N2O4S | [M+Na]+ | 1 |
| 12 | EMS67 | Antheraxanthin | 13.47 | 607.3956 | C40H56O3 | [M+Na]+ | 28 |
| 13 | EMS67 | Chlorophyllide | 14.02 | 615.2473 | C35H34MgN4O5 | [M+H]+ | 3 |
| 14 | EMS67 | Spectinomycin adenylate | 14.73 | 684.205 | C24H36N7O13P | [M+Na]+ | 7 |
| 15 | EMS67 | Palmitamide | 5.9 | 256.2659 | C16H33 | [M+H]+ | 9 |
| 16 | EMS67 | Benzyl Dimethylphosphonoacetate | 12 | 281.0555 | C11H15O5P | [M+Na]+ | 2 |

| | | | | | | | |
|---|---|---|---|---|---|---|---|
| 17 | EMS67 | Anthemis glycoside A | 14.96 | 832.2451 | $C_{39}H_{49}NO_{21}$ | [M+H-2H2O]+ | 26 |

PC abbreviated phosphatidylcholines, PI abbreviated glycerophosphoinositols; PG abbreviated glycerophosphoglycerols; PA abbreviated glycerophosphates.
